# Clinical Significance and Prognostic Effect of Serum 25-hydroxyvitamin D Concentrations in Critical and Severe Hand, Foot and Mouth Disease

**DOI:** 10.3390/nu9050478

**Published:** 2017-05-10

**Authors:** Hong-Xing Dang, Cheng-Jun Liu, Jing Li, Shi-Jiao Chen, Feng Xu

**Affiliations:** 1 Department of PICU, Children’s Hospital of Chongqing Medical University, Ministry of Education Key Laboratory of Child Development and Disorders, 136 Zhongshan No. 2 Road, Yu Zhong District, Chongqing 400014, China; danghx@hospital.cqmu.edu.cn (H.-X.D.); liucwd@163.com (C.-J.L.); lijingwangyi@126.com (J.L.); chengshijiao0808@163.com (S.-J.C.); 2 China International Science and Technology Cooperation base of Child development and Critical Disorders, 136 Zhongshan No. 2 Road, Yu Zhong District, Chongqing 400014, China; 3 Chongqing Engineering Research Center of Stem Cell Therapy, 136 Zhongshan No. 2 Road, Yu Zhong District, Chongqing 400014, China

**Keywords:** 25-hydroxyvitamin D, hand, foot and mouth disease, critical illness

## Abstract

Objective: To examine the association of serum 25-hydroxyvitamin D [25(OH)D] concentrations with critical and severe hand, foot and mouth disease (HFMD) and assess the clinical significance and prognostic effect of 25(OH)D concentrations in children with HFMD. Methods: This is a prospective observational study. The 138 children with HFMD were divided into common (49 cases), severe (52 cases), and critical (37 cases) HFMD groups. Another 59 healthy children undergoing outpatient medical examinations during the same period were chosen as the control group. Serum 25(OH)D concentrations were measured in all the subjects, and each group was subdivided by serum 25(OH)D concentration into 25(OH)D normal (≥30 ng/mL); insufficiency (20–29.9 ng/mL), and deficiency (<20 ng/mL) groups. The pediatric critical illness score (PCIS) was recorded for the critical and severe HFMD group upon admission to the pediatric intensive care unit (PICU). Children with critical and severe HFMD were also monitored for blood lactate (LAC), serum calcium ions (Ca++), D-dimer (DD), lactate dehydrogenase (LDH), and creatine kinase-MB (CK-MB) levels; the incidences of brainstem encephalitis, neurogenic pulmonary edema, and circulatory failure; and the 14-day mortality rate. Results: Serum 25(OH)D concentrations were generally low in all groups. The critical HFMD group showed a significantly lower serum 25(OH)D mean concentration (20.0 ± 8.4 ng/mL) and a higher proportion of deficiency (18%) compared with the control group (28.1 ± 6.6 ng/mL, 8%), common (29.5 ± 8.1 ng/mL, 10%) and severe (31.9 ± 9.7 ng/mL, 8%) HFMD groups (*p* < 0.05). In the critical and severe HFMD groups, the 25(OH)D deficiency group had lower PCISs than the 25(OH)D normal and insufficiency groups (*p* < 0.05); and had higher values than the latter two groups for LAC, LDH, CK-MB and DD; and the incidences of brainstem encephalitis, neurogenic pulmonary edema, circulatory failure, and mortality (*p* < 0.05). The death group showed significantly lower serum 25(OH)D concentrations and PCISs than the survival group (*p* < 0.05) and had higher LAC, LDH, CK-MB and DD levels and higher incidences of brainstem encephalitis, neurogenic pulmonary edema, and circulatory failure (*p* < 0.05). Logistic regression analysis revealed that the serum 25(OH)D concentration was an independent factor that influenced mortality in children with critical and severe HFMD. Conclusions: In this study, we find the serum 25(OH)D concentrations are substantially reduced in children with critical and severe HFMD and are associated with the severity of HFMD. The serum 25(OH)D concentrations may have clinical value for determining the progression of critical HFMD and predicting the risk of death. Further evidence is needed before it can be stated that 25(OH)D concentrations have clinical value in HMFD diagnosis.

## 1. Introduction

Hand, foot and mouth disease (HFMD) has become an endemic childhood disease in East and Southeast Asia [1]. Coxsackievirus A16 (CA16) and human enterovirus 71 (EV71) are the most common cause of HFDM and have often circulated alternatively or together. EV71 infection has been reported to be associated with many outbreaks of HFMD worldwide and appear to be increasing in size and severity, especially the great outbreaks that occurred in the Asia-Pacific region. Critical and severe HFMD are associated with rapid changes and a poor prognosis. In particular, children with HFMD caused by an EV71 infection may die from the sudden onset of brainstem encephalitis, neurogenic pulmonary edema, pulmonary hemorrhage, and respiratory and circulatory failure [2]. This virus is considered a critical emerging public health threat and still has no effective antiviral drug. Most cases of critical and severe HFMD that resulted in hospitalization for severe complications occurred in young children. But the mechanism by which EV71 causes severe central nervous system complications remains unclear. Studies suggest that may result from a combination of pathological immune responses and direct viral effects, but no effective treatment is currently available and no biomarker that can be used as an early warning for critical HFMD. Chongqing (southwest of China) is a high incidence area of severe and critical HFMD [3], but also a high incidence of vitamin D (VitD) deficiency and insufficiency [4]. Studies have already suggested that the deficiency of (VitD) was found in some critical and severe disease [5], and in our clinical practice, we also found some critical HFMD children with a very low serum concentration of 25-hydroxyvitamin D [25(OH)D]. This aroused our interest. As such, we hypothesized whether the concentrations of vitamin D were related to the severity of HFMD. VitD has extensive biological effects in addition to regulating the calcium and phosphorus balance [5]. Increasing research has been conducted concerning VitD, particularly in terms of its role in regulating immunity, attenuating inflammatory responses, and protecting the central nervous system [6]. Given the regulatory role of VitD in infectious immunity and inflammatory responses, we speculated that this vitamin may be involved in the progression of critical and severe HFMD. In the present study, we measured the 25(OH)D concentrations in children with HFMD and assessed the clinical significance and prognostic effect of 25(OH)D concentrations in order to provide a reference for clinical diagnosis and treatment.

## 2. Methods and Materials

### 2.1. Study Subjects

**Figure 1:**
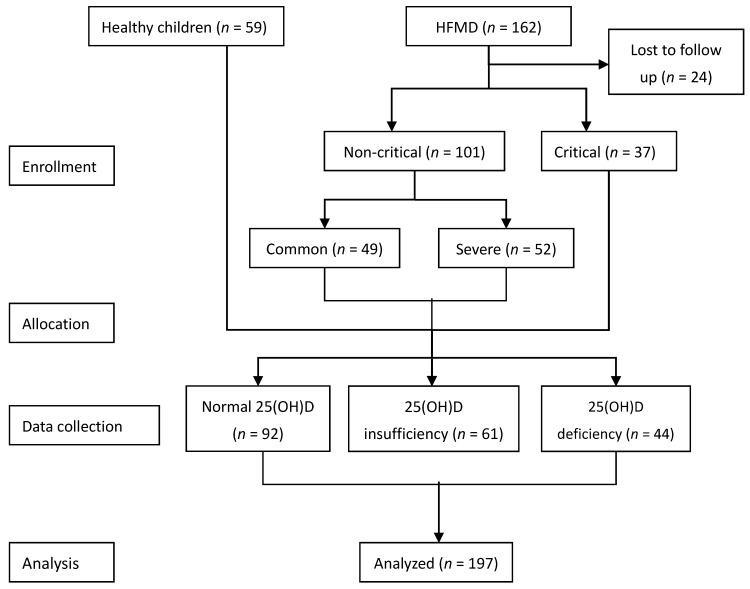
Research roadmap.

A prospective observational study was conducted on children with HFMD who were admitted to the Children’s Hospital of Chongqing Medical University of China in June 2015 to September 2016 (Figure 1). The subjects included common cases admitted to the Department of Infectious Disease and critical and severe cases admitted to the pediatric intensive care unit (PICU). Healthy children undergoing outpatient medical examinations during the same period were selected as the control group. All subjects were selected from each quarter during the study period by a random number table. The study was approved by the Ethics Committee of the Children’s Hospital of Chongqing Medical University of China (Approval No.: (2015) Ethics Review (Research) No. (92)), and registered at the Chinese Clinical Trial Registry (http://www.chictr.org.cn/enIndex.aspx; Registration No.: ChiCTR-OOC-15007152). All guardians and (or) patients were informed and signed the consent form.

### 2.2. Inclusion and Exclusion Criteria

Inclusion criteria for HFMD: (1) Both the diagnosis and clinical classification complied with the Diagnosis and Treatment Guidelines for Hand-Foot-Mouth Disease (2010 Edition) developed by the Ministry of Health of the People’s Republic of China [7]; (2) The patients were children (0–14 years old). Exclusion criteria: (1) Patients who were previously diagnosed with VitD deficiency or currently undergoing VitD supplementation; (2) Patients who had recently used high-dose glucocorticoids or other drugs that affect VitD metabolism; and (3) Patients who suffered from parathyroid or immunodeficiency disease, rickets, diabetes, serious liver and kidney function impairment, or other diseases affecting VitD metabolism.

### 2.3. Data Collection

(1) Blood specimens (3 mL each) were collected from the subjects. The specimens were collected from the healthy children during their physical examination, from the children with common HFMD on the day of admission to the Department of Infectious Diseases, and from the children with critical and severe HFMD upon their admission to the PICU. All intravenous blood collections were performed at least 6 h after the last meal. Each specimen was immediately centrifuged to obtain 1–2 mL of serum. The samples were frozen at −80 °C and sent to a third party for the quantitative analysis of 25(OH)D. Meanwhile, the specimens taken from the children with critical and severe HFMD underwent analyses of blood lactate (LAC), serum-free calcium ions (Ca++), D-dimer (DD), lactate dehydrogenase (LDH), and creatine kinase-MB (CK-MB); (2) The general information of all subjects was collected, including age, gender, weight, height, address, telephone number, vital signs, past medical history, and medication history; (3) When the children with critical and severe HFMD were admitted to the PICU, their pediatric critical illness score (PCIS) was evaluated by two attending physicians independently, and the results were averaged. The total possible score was 100 points, and the severity of HFMD was ranked as follows: >80 points, non-critical; 71–80 points, critical; and ≤70 points, extremely critical [8]. The PCIS scorers were unaware of the results of 25(OH)D analysis or the patients’ outcomes. Additionally, the incidences of brainstem encephalitis, neurogenic pulmonary edema, and heart failure were recorded, and their correlations with the serum 25(OH)D concentrations were analyzed; (4) All patients were monitored for 14 days, with the 14-day outcome as the end point. Patients hospitalized for fewer than 14 days were followed-up via telephone. Patient survival and death were recorded.

### 2.4. Measurement Method and Range of Reference Values

The serum 25(OH)D was analyzed using liquid chromatography-tandem mass spectrometry. The pretreatment method of liquid–liquid extraction was used in the project, including a quality control. The experimental standard and internal standard were purchased from SIGMA (St. Louis, MO, USA). The quality control was purchased from RECIPE (Munich, Germany). In order to ensure the accuracy of the method, we used the standard substance SRM972 (from American Standards Committee) to verify the accuracy of the 25(OH)D detection method. As defined by the US Endocrine Society’s clinical practice guideline [9], the serum 25(OH)D concentrations were classified as follows: ≥30 ng/mL, normal; 20–29.9 ng/mL, insufficiency; and <20 ng/mL, deficiency.

### 2.5. Statistical Methods

All data were analyzed using SPSS Statistics 19.0 software (IBM, Chicago, IL, USA) and checked for normality. Normally, distribution data are reported as M ± S, and non-normal distribution data are reported as median (quartile) [M(QR)]. These data were subjected to the t-test, rank-sum test, and analysis of variance. Count data were analyzed using the chi-square test. The relationships between the serum 25(OH)D and various factors (Age, body mass index (BMI), 25(OH)D, LDH, LAC, CK-MB, DD, Ca++ and PCIS) were examined using Spearman correlation analysis and the independent prognostic factor was determined using logistic regression analysis. A *p*-value of less than 0.05 was considered statistically significant.

## 3. Results

### 3.1. General Condition of the Selected Subjects 

A total of 162 children with HFMD were initially selected. Of these, 24 cases were associated with incomplete data or loss to follow-up. Thus, 138 hospitalized patients with HFMD were finally included in the study. Another 59 healthy children undergoing outpatient medical examinations during the same period were included. Differences in age, gender, and BMI were not statistically significant between the healthy control, common HFMD, severe HFMD and critical HFMD groups (*p* > 0.05; Table 1).

### 3.2. Comparison of 25(OH)D Concentrations and the Incidence of 25(OH)D Deficiency and Insufficiency among Groups

Throughout the study period (about 15 months), the serum 25(OH)D concentrations of all groups were generally low. The total incidences of 25(OH)D deficiency and insufficiency were 49% in the healthy control group, 53% in the common HFMD group, 37% in the severe HFMD group, and 84% in the critical HFMD group. The serum 25(OH)D concentration was significantly lower and the proportion of 25(OH)D deficiency patients was significantly higher in the critical HFMD group (20.0 ± 8.4 ng/mL, 47%) compared with the healthy control (28.1 ± 6.6 ng/mL, 8%), common HFMD (29.5 ± 8.1 ng/mL, 20%), and severe HFMD groups (31.9 ± 9.7 ng/mL, 15%) (*p* < 0.05). However, no significant difference was observed in the serum 25(OH)D concentrations or the proportion of 25(OH)D deficiency and insufficiency between the non-critical (common and severe) HFMD and control groups (*p* > 0.05; Table 2). 

### 3.3. Comparison of 25(OH)D Concentrations and Prognostic Relationships between Subgroups in the Critical and Severe HFMD Group

In this study, compared with the 25(OH)D normal and insufficiency groups, the 25(OH)D deficiency group had significantly higher LAC, LDH, CK-MB and DD levels; higher incidences of brainstem encephalitis, neuronal pulmonary edema, and circulatory failure; and higher mortality rates (*p* < 0.05). Furthermore, the 25(OH)D deficiency group had lower PCIS than the other two groups (*p* < 0.05). These indicators showed no significant differences between the 25(OH)D insufficiency and normal groups (*p* > 0.05). The difference in the serum Ca++ concentration was not statistically significant among the three groups (*p* > 0.05; Table 3).

### 3.4. Comparison of Death and Survival in Children with Critical and Severe HFMD

Nineteen of the 89 children with critical and severe HFMD died, for a mortality rate of 21.3%. A comparison analysis revealed that the death group had markedly lower serum 25(OH)D and PCIS levels but higher LAC, LDH, CK-MB and DD levels compared with the survival group; the group differences were statistically significant (*p* < 0.05). No significant difference was observed between the two groups in terms of Ca++ level (Table 3). All of the children in the death group suffered brainstem encephalitis, neuronal pulmonary edema, and circulatory failure. The two groups showed no significant difference in age or BMI (*p* > 0.05; Table 4).

### 3.5. Logistic Regression Analysis of the Prognostic Effect of 25(OH)D in Children with Critical and Severe HFMD

Logistic regression was performed for the age, BMI, 25(OH)D, LDH, LAC, CK-MB, DD, Ca++ and PCIS levels to determine the independent factors affecting the prognosis of children with critical and severe HFMD. The results show that 25(OH)D, DD and PCS are independent factors affecting the 14-day mortality in children with critical and severe HFMD (*p* < 0.05; Table 5). 25 (OH) D and PCIS are protective factors, and DD is a risk factor.

## 4. Discussion

HFMD is prevalent in infants and children under the age of five. Common HFMD has mild clinical symptoms that mainly manifest as fever, mouth sores, and skin rashes on the hands and feet. The disease course is often self-limited. A minority of children can rapidly progress to severe or even critical HFMD, which is accompanied by serious complications such as brainstem encephalitis, neurogenic pulmonary edema, and circulatory failure [2]. Once the condition becomes critical, mortality rates can be significantly higher. In the present study, we found that children of a younger age had a relatively high incidence of critical and severe HFMD, which may be related to the imperfect development of their autoimmune systems [10,11]. The pathogenesis of critical and severe HFMD mainly involves viral infection, uncontrolled inflammatory response, and autoimmune disorders leading to impairment of the nervous system and heart and lung function. In addition to stabilizing the calcium and phosphorus balance, VitD has a wide range of ectosteal biological effects. VitD receptors and 25(OH)D-hydroxylase, a key enzyme in VitD metabolism, are widely present in the cells of the human small intestine, parathyroid, pancreas, bone, colon, myocardium, placenta, pituitary gland, arteries, and other tissues. Preliminarily, 2776 binding sites have been found in the human genome using microarray technology. Transcriptome-wide analysis indicated that, depending on the cell type, more than 1000 genes change their mRNA expression after a 24 h stimulation with 1,25(OH)_2_D_3_ [12]. These alterations form the foundation for the pleiotropic biological effects of VitD. 

Recent studies demonstrated that serum VitD deficiency is an independent risk factor for mortality among children in the ICU [13,14]. A study [15] indicated that VitD deficiency affects the catecholamine system and exacerbates the instability of the cardiovascular system. Moreover, animal experiments have shown that the knockout of the VitD receptor gene can lead to symptoms such as cardiac hypertrophy, hypertension, and increased water infiltration in mice [16]. Lee et al. [17] also suggested that because VitD deficiency results in increased mortality among critically ill children in the ICU, the regulatory role of VitD in the infective immunity and inflammatory response is indicative of its involvement in the pathogenesis of sepsis and its clinical value [18,19]. Recently, a multicenter study of 568 children with sepsis showed that VitD deficiency is an important predictor of the emergence of sepsis in children, and that VitD insufficiency and deficiency can increase the risk of death in children with sepsis [20]. The progression of severe to critical HFMD is associated with inflammatory storm and autoimmune disorders induced by the viral activation of inflammatory cytokines in vivo, which is in common with severe sepsis.

The 25(OH)D concentration varies in different latitudes according to the duration of sunlight exposure. VitD deficiency or insufficiency is prevalent in Chinese children, and is found in 63.4% of children aged 0–14 years [21]. Our study also found that VitD deficiency or insufficiency was relatively serious in the healthy children undergoing physical examination in Chongqing. Children’s metabolism is fast and needs extra energy to grow. A general diet may not meet the daily VitD requirement [22]. Chongqing is located in Southwest China and the local economy is relatively underdeveloped. Coupled with lack of knowledge of proper health care, many children do not receive VitD supplements. These reasons may be responsible for the high incidence of VitD deficiency and insufficiency of children in Chongqing. In this study, which were up to 83.8% in children with critical HFMD, indicating that they had more serious conditions than the healthy control and common HFMD groups did. 

Currently, it is generally thought that the cutoff between VitD deficiency and insufficiency is based on the function of VitD in bone health and muscle, whereas no consideration is given to the endosteal effect of VitD. Therefore, it is impossible to determine whether the required VitD levels in different organs to maintain the best functional state differ or are identical [23]. Lee [24] proposed that VitD deficiency is essentially an imbalance between the supply and the demand from the tissues. Huge pathophysiological changes occur in the bodies of children with critical HFMD. Because of an increase in VitD wasting, and reduced intake and synthesis at the histiocyte level [24]. The above definition of VitD deficiency for the general population may not apply to critically ill children. We need to constantly explore the definition for the critically ill population in practice. In the present study, however, it was clear that the lower the serum 25(OH)D concentrations, the higher the incidence of serious complications and mortality in children with HFMD.

The PCIS is the most commonly used scoring system for disease severity and mortality risk in children in the PICU. Our study showed that among the children with critical and severe HFMD who were admitted to the PICU, the 25(OH)D deficiency group had a lower PCIS than the 25(OH)D insufficiency and normal groups did. That is, in this study, the lower the 25(OH)D concentration is, the lower the PCIS score, and possibly the higher the disease severity and mortality risk in children with critical and severe HFMD. Furthermore, we also examined LAC, LDH, CK-MB, DD and Ca++ levels in children with critical and severe HFMD. The results showed that the serum 25(OH)D concentration was negatively correlated with Ca++ in these children; but the lower the 25(OH)D concentration was, the higher the incidences of brainstem encephalitis, neurogenic pulmonary edema, circulatory failure, and mortality. These findings further suggest that maybe there is a high correlation between the serum 25(OH)D and the prognosis of children with critical and severe HFMD. In the present study, the serum Ca++ levels showed no significant difference among the children with critical and severe HFMD, regardless of whether they were in the 25(OH)D normal, insufficiency, or deficiency group. This indicates that serum 25(OH)D may have no close association with serum Ca++ in children with HFMD. The regulation of the serum Ca++ levels may not entirely rely on serum 25(OH)D and is not closely related to the prognosis of children with HFMD.

The results of this study show that the serum 25(OH)D concentration is an independent factor affecting the 14-day mortality rate of children with critical and severe HFMD. A reduction in the serum 25(OH)D concentration may aggravate the condition of critical and severe HFMD patients and increase the risk of death in children. Perhaps, these results provide a warning about disease severity and are likely to play a role in promoting disease progression, thereby affecting the prognosis of children with HFMD. Otherwise, according to this study, combining the serum 25(OH)D concentrations with the PCIS and other indicators, such as LAC, LDH, CK-MB and DD, may help to evaluate the condition of critical and severe HFMD, and monitoring the serum 25(OH)D concentrations in children with critical and severe HFMD may assist in determining the progression and prognosis of the disease. But this is only one small observational study; detailed, larger samples and follow-up studies are needed to provide more evidence before any guidelines or the specific role of VitD in HFMD is determined.

Research on VitD and sepsis has increased in recent years [25]. The biological pleiotropy of VitD shows bright prospects for the treatment of sepsis [26,27,28]. However, little research has been conducted, particularly concerning critical and severe HFMD. The present study had a small sample size and a short study period, with a lack of dynamic monitoring; moreover, serum 25(OH)D concentration showed great variations in vivo. Therefore, it is necessary to expand the sample size and extend the study time to further clarify the mechanism of 25(OH)D deficiency and HFMD progression, and to determine whether VitD supplements can reduce mortality and slow the progression of severe and critical HFMD. 

## Figures and Tables

**Table 1:** General data of the study subjects.

Group	Healthy Control		Non-Critical HFMD				Critical HFMD		* *p*
			Common		Severe				
Gender (*n*)	Male	Female	Male	Female	Male	Female	Male	Female	0.83
	31	28	23	26	29	23	20	17	
Age (month)	29 ± 13		30 ± 11		29 ± 13		26 ± 11		0.55
BMI	16.5 ± 1.8		16.7 ± 2.3		15.9 ± 1.9		15.9 ± 2.1		0.15

* Comparison among groups, *p* > 0.05. HFMD denotes hand, foot and mouth disease.

**Table 2:** Serum concentrations of 25-hydroxyvitamin D [25(OH)D] and incidences of 25(OH)D deficiency and insufficiency in different groups.

	Healthy Control	Common HFMD	Severe HFMD	Critical HFMD
*n*	59	49	52	37
25(OH)D (ng/mL)	28.1 ± 6.6	29.5 ± 8.1	31.9 ± 9.7	20.0 ± 8.4
25(OH)D > 30 ng/mL, *n* (%)	30(51)	23(47)	33(64)	6(16)
25(OH)D 20–29.9 ng/mL, *n* (%)	21(36)	16(33)	11(21)	13(35)
25(OH)D ≤ 20 ng/mL, *n* (%)	8(13)	10(20)	8(15)	18(49)
* *p*	<0.001	0.004	<0.001	

* *p*: Critical HFMD vs. Healthy Control, Common HFMD and Severe HFMD respectively. HFMD denotes hand, foot and mouth disease.

**Table 3:** Comparison of subgroups of children with critical and severe hand, foot and mouth disease.

Group	Normal 25(OH)D (*n* = 33)	25(OH)D Insufficiency (*n* = 25)	25(OH)D Deficiency (*n* = 31)	* *p*
Age (month)	31 ± 12	25 ± 9	27 ± 13	0.95
BMI	16.1 ± 1.7	15.7 ± 1.9	16 ± 12	0.93
25(OH)D (ng/mL)	36 ± 6.2	24.8 ± 3.0	13.6 ± 3.6	<0.001
LAC (mmol/L)	2.6 ± 2.8	4.3 ± 3.4	7.2 ± 4.3	0.004
LDH (U/L)	600 ± 600	500 ± 200	1300 ± 900	<0.001
CK-MB (μg/L)	3.4 ± 2.1	3.4 ± 1.7	10.9 ± 10.2	0.001
DD (mg/L)	1.7 ± 2.0	2.1 ± 1.4	4.8 ± 4.9	0.02
Ca++ (mmol/L)	1.0 ± 0.3	1.2 ± 0.2	1.0 ± 0.3	0.06
PCIS	85 ± 11	72 ± 8	66 ± 11	0.02
Brainstem encephalitis (case)	3	6	16	0.04
Neurogenic pulmonary edema (case)	2	5	16	0.02
Circulatory failure (case)	2	4	15	0.01
Death (case)	2	4	13	0.04

* *p*: 25(OH)D deficiency vs. insufficiency groups. (LAC, blood lactate; LDH, lactate dehydrogenase; CK-MB, creatine kinase-MB;DD, D-dimer; Ca++, serum calcium ions; PCIS, pediatric critical illness score).

**Table 4:** Comparison of the death and survival groups of children with critical and severe hand, foot and mouth disease.

Prognostic Group	Survival (*n* = 70)	Death (*n* = 19)	*p*
Age (month)	28 ± 12	27 ± 12	0.79
BMI	15.7 ± 1.9	16.4 ± 2.2	0.19
25(OH)D (ng/mL)	30.0 ± 9.7	15.6 ± 6.7	<0.001
LAC (mmol/L)	3.3 ± 3.0	9.5 ± 3.5	<0.001
LDH (U/L)	500 (300–700)	1700 (500–2600)	0.01
CK-MB (μg/L)	2.6 (1.4–4.0)	14 (4–21)	<0.001
DD (mg/L)	1.7 ± 1.7	7.2 ± 5.0	<0.001
Ca++ (mmol/L)	1.06 ± 0.3	1.11 ± 0.3	0.60
PCIS	79 ± 11	60 ± 9	<0.001

*p*: Compared with the survival group. (LAC, blood lactate; LDH, lactate dehydrogenase; CK-MB, creatine kinase-MB; DD, D-dimer; Ca++, serum calcium ions; PCIS, pediatric critical illness score).

**Table 5:** Logistic regression analysis of factors affecting mortality in critical and severe hand, foot and mouth disease.

Indicator	Regression Coefficient (β)	*p*	OR	95% Confidence Interval
Age	−0.38	0.06	0.68	0.46–1.02
BMI	−0.78	0.19	0.46	0.14–1.46
25(OH)D *	0.71	0.045	2.03	1.02–4.06
LDH	0.01	0.06	1.01	1.00–1.01
LAC	−0.74	0.07	0.48	0.22–1.07
CK-MB	0.24	0.30	1.27	0.81–1.99
DD *	−1.22	0.04	0.30	0.09–0.93
Ca++	−4.48	0.15	0.01	0.00–4.69
PCIS *	0.39	0.03	1.48	1.04–2.10

* *p* < 0.05 (LAC, blood lactate; LDH, lactate dehydrogenase; CK-MB, creatine kinase-MB; PCIS, pediatric critical illness score).
